# Gas-Phase Formation of Trioxy Acid via OH-Initiated Aldehyde Oxidation under Atmospheric Conditions

**DOI:** 10.1021/acs.jpclett.5c03582

**Published:** 2026-01-16

**Authors:** Emelda Ahongshangbam, Avinash Kumar, Shawon Barua, Melissa Meder, Matti Rissanen, Nanna Myllys

**Affiliations:** ‡ Department of Chemistry, 3835 University of Helsinki, Helsinki 00014, Finland; ¶ Institute for Atmospheric and Earth System Research, University of Helsinki, Helsinki 00014, Finland; § Aerosol Physics Laboratory, Tampere University, Tampere, 33720, Finland

## Abstract

The formation of a new carbonyl compound containing three linearly bonded oxygen atoms attached to the carbonyl carbon with a chemical formula of RC­(O)­O_3_H (trioxy acid) has been detected. The trioxy acid is formed in the reaction of benzaldehyde-derived acyl peroxy radicals and OH radicals under atmospherically relevant conditions. We employed flow reactor experiments with chemical ionization mass spectrometry, supported by quantum chemistry, to investigate the competitive reaction channels of acyl peroxy radicals formed during OH-initiated oxidation of aldehydes. While the role of trioxy acid in oxidation chemistry and cluster formation is not understood, this study provides insights in their formation and stability under atmospherically relevant conditions.

## Graphical abstract

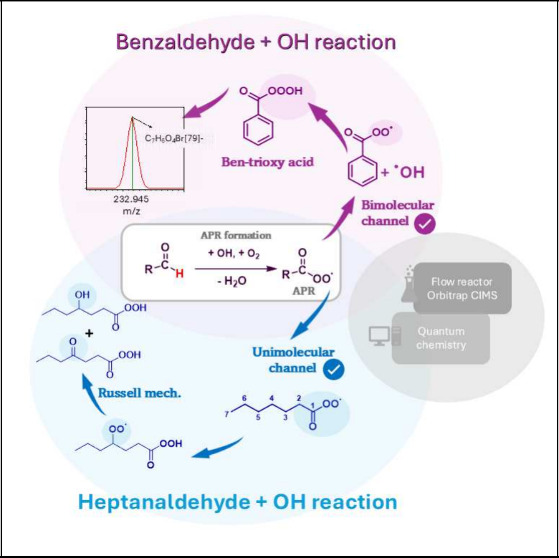

Gas-phase organic compounds containing three oxygen atoms bonded linearly to one another are primarily regarded as unstable and highly reactive. They are coined as trioxides, generally formed as an intermediate in the bimolecular reaction of organic peroxy radicals (RO_2_) and hydroxyl radicals (OH).^1,2^ In the aqueous phase, these trioxides are formed during the ozonolysis of organic compounds at low temperatures in organic solvents.^3^ Despite being highly decomposable into smaller radicals, experimental detection of hydrotrioxides in the gas-phase has been demonstrated recently by Berndt et al.^1^ and Caravan et al.^2^ In the study of Berndt et al.,^1^ the detected hydrotrioxides are formed from trimethylamine, dimethyl sulfide, α-pinene, toluene or 1-butene derived peroxy radicals. The kinetic analysis confirmed that the rate coefficient of this bimolecular reaction via the RO_2_ + OH mechanism is approaching the collision limit.^4,5^

Similarly, acyl peroxy radicals (APRs) are notable species of peroxy radicals containing a carbonyl group (RC­(O)­O_2_) and are reported to have concentrations up to 10^8^ cm^–3^ in the atmosphere.^6,7^ Such radical species are reactive and can even initiate oxidation of unsaturated hydrocarbons.^8,9^ APRs are dominantly formed by the hydrogen abstraction reaction of aldehydes by OH radicals or by the photolysis reactions of ketones, followed by the addition of molecular oxygen. They can undergo numerous unimolecular and bimolecular reactions, depending on the chemical system and atmospheric conditions. Under low NOx conditions, APRs react with HO_2_ forming peracids (RC­(O)­O_2_H), or with other peroxy radicals through recombination channels. Recently, several studies^10–13^ theoretically investigated the unimolecular H-shift and endoperoxide ring formations of several types of APR. The APR structures, which are small or rigid, tend to have slow unimolecular reactions, and they are prone to bimolecular reactions, including the oxidation of unsaturated hydrocarbons.^8,13^ Moreover, APR + APR dimerization reactions are proposed to yield low-volatility products through mechanisms analogous to RO_2_ + RO_2_ reactions.^14,15^ Owing to the higher reactivity of APRs than many peroxy radicals, distinct reaction pathways may govern their atmospheric chemistry. Building upon the critical role of APRs in oxidation chemistry, coupled with the recent discovery of hydrotrioxides formed via the RO_2_ + OH mechanism,^1^ this study focuses on the detection and detailed characterization of closed-shell products generated through the APR + OH mechanism, where APRs are investigated from two types of aldehydes. This investigation not only emphasizes the significant role of APRs as oxidants but also deepens the understanding of the chemical pathways leading to complex oxidation products associated with the aldehydes under low NOx conditions.

In this study, we investigated OH-initiated aldehyde oxidation using two types of aldehydes bearing structurally and chemically distinct R-groups but identical carbon numbers. To represent aromatic and long-chain aliphatic R-groups, benzaldehyde and heptanaldehyde were selected as the respective aldehyde precursors. APRs were produced experimentally by the reaction of aldehydes with OH radicals in a flow tube. The OH radical abstracts an aldehydic hydrogen to form acyl radicals. Subsequently, molecular oxygen adds to the radical center, resulting in the formation of APR. The general mechanism of the reaction producing APR and a bimolecular product (trioxy acid) is presented in Figure 1.

**Figure 1:**
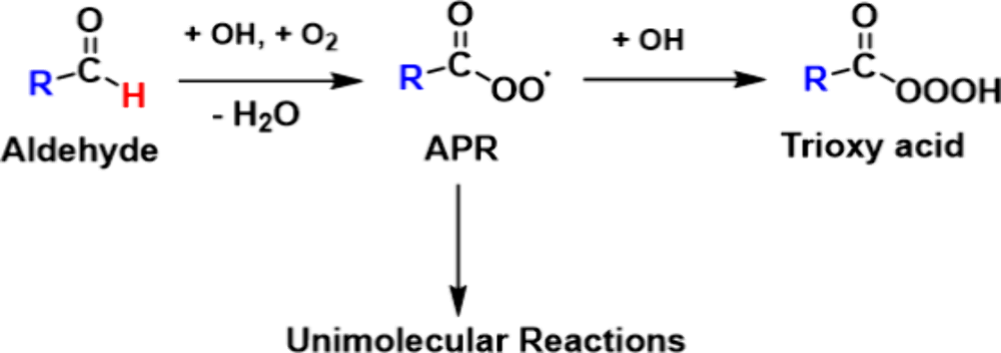
General reaction mechanism showing acyl peroxy radical (APR) formation and potential subsequent reactions investigated experimentally in this study.

Figure 2 presents the mass spectrum of the O_4_-products resulting from the OH-initiated oxidation of benzaldehyde. It is noteworthy that bromide ionization cannot be used to detect any mass signal corresponding to benzoyl peroxy radicals (ben-APR). The reason could be the short lifetime of ben-APR and the absence of a partial positive charge in the ben-APR molecules. Moreover, the lack of hydrogen bonding functionalities in the ben-APR molecule prevents the effective adhesion of bromide ions, thereby affecting detection as indicated by Gibbs binding energy of −0.04 kcal mol^–1^. The red and blue peaks shown in the mass spectra correspond to the product cluster signals of bromide isotopes Br[79] and Br[81], respectively. The highest intensity peak (see red line) with the exact mass-to-charge ratio at 232.945 Th matches the formed C_7_H_6_O_4_*Br­[79]^−^ cluster. A peak (blue line) corresponding to the isotopic cluster, i.e, C_7_H_6_O_4_*Br­[81]^−^ is also observed with the nominal mass-to-charge ratio at 234.943 Th.

**Figure 2:**
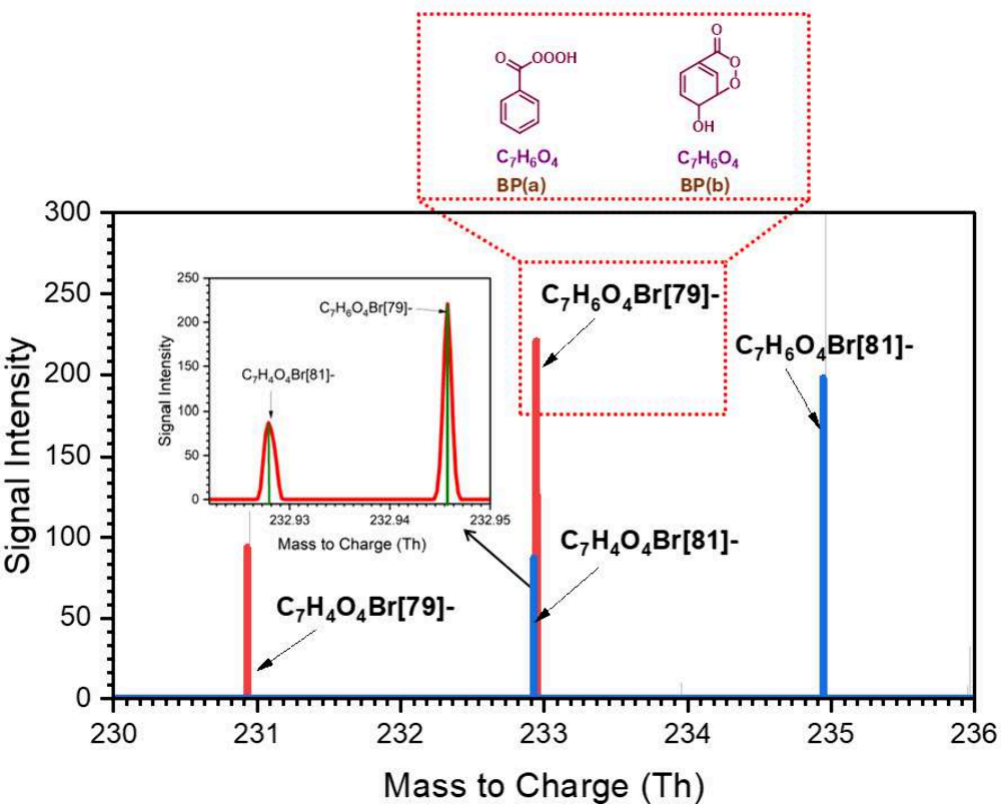
Bromide chemical ionization mass spectrum of O_4_-system (or products) in the OH-initiated benzaldehyde oxidation. The two potential molecular structures of the chemical formula C_7_H_6_O_4_ are presented within the dashed red box (see Figure 2 in the Supporting Information for formation mechanisms). The zoomed-in spectrum containing the signals of C_7_H_6_O_4_Br­[79]^−^ and C_7_H_4_O_4_Br­[81]^−^ are presented for more clarity. Note: The red and blue peaks are the mass spectra of the corresponding cluster peak with two isotopes of bromide ions, Br[79]- and Br[81]-, respectively.

The two potential molecular structures of the molecular formula (C_7_H_6_O_4_) are distinctively highlighted in the dashed red box. The structures representing the proposed products, namely, BP­(a) and BP­(b), arising from the potential chemical transformation of ben-APR, are shown in detail in Figure 2 in the Supporting Information. The structure BP­(a) corresponds to the benzaldehyde-derived trioxy acid (ben-trioxy acid), consisting of three bonded oxygen atoms. It is formed in the bimolecular reaction of the ben-APR + OH mechanism. Our computations estimate the association rate between ben-APR and OH to be 8.0 × 10^–10^ cm^3^ molecule^–1^ s^–1^. The reaction is found to be barrierless and highly exothermic. Moreover, a recent study by Chen et al.^16^ theoretically investigated the general mechanism of such reactions, particularly for the CH_3_C­(O)­O_2_ + OH, and observed a barrierless formation with a rate coefficient of 1.8 × 10^–10^ cm^3^ molecule^–1^ s^–1^. On the other hand, the structure BP­(b) is a closed-shell hydroxy-functionalized product formed via unimolecular endoperoxide ring formation reaction of ben-APR, followed by the addition of O_2_ to the radical center. The second-generation peroxy radicals can react with each other through the well-known Russell mechanism.^17^ This mechanism involves intermolecular hydrogen transfer and leads to structure BP­(b) and the corresponding carbonyl product (with a chemical formula of C_7_H_4_O_4_) in an equal amount (see Figure 2 in the Supporting Information).

To confirm the molecular structures of C_7_H_6_O_4_ and C_7_H_4_O_4_, we conducted additional hydrogen–deuterium exchange (H/D) experiments by adding deuterated water (D_2_O) to the gas stream. Deuterium atoms present in D_2_O molecules (in excess) replace labile hydrogens (in particular, from −OH, -OOH or -OOOH groups) and form products with one-unit mass shift for each −OH, -OOH or -OOOH group. The mass spectral plot of these hydrogen–deuterium exchange reactions is provided in Figure 7 in the Supporting Information. The result shows that exactly one −OH group is present in the C_7_H_6_O_4_ product, which agrees with both proposed structures, BP­(a) and BP­(b). In order to differentiate these two structures, we need to remember that the Russell mechanism produces two products: the hydroxyl compound C_7_H_6_O_4_ with one labile hydrogen and the carbonyl compound C_7_H_4_O_4_ without any labile hydrogen. Our H/D experiments show that the signal corresponding to C_7_H_4_O_4_ shifts by one-unit mass when D_2_O is added, meaning it contains one labile hydrogen, and therefore, the O_4_-products formation via the Russell mechanism can be excluded.

Moreover, our recent calculations show that ben-APR has very slow unimolecular reactions; the cyclization rate coefficient is on the order of 10^–6^ s^–1^.^12^ This means that ben-APR reacts bimolecularly (more details in †ESI S9). Therefore, with high confidence, we can exclude the structure BP­(b), and conclude that the structure C_7_H_6_O_4_ is trioxy acid BP­(a), which is formed in the bimolecular reaction of ben-APR + OH mechanism. In addition, the detection of ben-trioxy acid by bromide ions is supported by their Gibbs binding energy of −8.9 kcal mol^–1^. Furthermore, the detection of the bimolecular reaction product (ben-trioxy acid) is also confirmed by experiments with nitrate chemical ionization mass spectrometry (nitrate-CIMS), where the oxidant OH radicals are produced in situ by TME + O_3_ reaction (TME = tetramethylethylene) and is shown in Figure 9 in the Supporting Information. For APR structures possessing sufficiently small or rigid structures, trioxy acid formation could be more likely due to slow unimolecular reactions. For instance, anthropogenic aldehydes often contain aromatic rings, which make their structure rigid, thus acting as potential trioxy acid precursors. Whereas green leaf aldehydes have a long carbon chain, allowing rapid unimolecular reactions. Additionally, at high NOx conditions, PAN formation is limiting other potential bimolecular reactions of APRs.

Our quantum chemical calculations indicate that ben-trioxy acid is stable enough to be detected in our experiments, as the possible thermal decomposition routes (T = 298 K) are relatively slow. Computed ben-trioxy acid decomposition rate coefficient of 2.2 × 10^–1^ s^–1^ into corresponding alkoxy and HO_2_ radicals indicates a unimolecular lifetime of several seconds. Possible explanation for this is that the intramolecular hydrogen bonding induces the formation of a complementary six-membered ring structure in addition to the existing aryl ring and that the carbonyl group in ben-trioxy acid participates in extended conjugation, enhancing the resonance stabilization (see Figure 3). Recently, Berndt et al.^1^ also have shown that the hydrotrioxide structure ((HOOCH_2_)_2_NCH_2_OOOH) is highly stabilized due to the presence of three intramolecular hydrogen bonds. In contrast, such stabilization is absent in aliphatic counterparts, such as in CH_3_C­(O)­OOOH ace-trioxy acid, which, according to theoretical results by Chen et al.,^16^ forms as an intermediate and decomposes rapidly into acetic acid. Also, isoprene-derived hydrotrioxides are shown to decompose rapidly through the alkoxy pathway (decomposition rate coefficients in the order 10^–1^ s^–1^).^18^ These differences highlight that the formation and decomposition of trioxy acids are likely to be strongly structure-dependent and distinctive.

**Figure 3:**
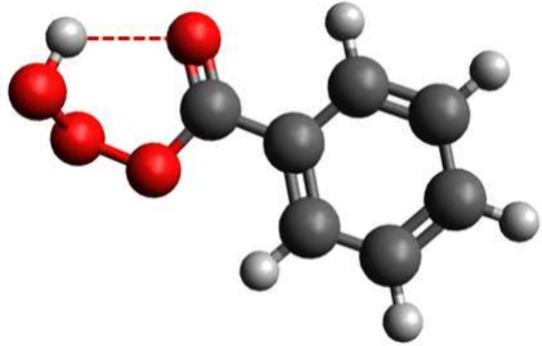
Global minimum structure of benzaldehyde-derived trioxy acid formed in the OH-initiated benzaldehyde oxidation. Color coding: light gray is hydrogen, dark gray is carbon, and red is an oxygen atom.

In addition to benzaldehyde, we studied the possible trioxy acid formation of an aldehyde with long-chain aliphatic R-group heptanaldehyde (C_7_H_14_O) containing the same number of carbon atoms in order to compare the behavior of the same size APRs. Figure 4 illustrates the mass signals of two chemical species along with their corresponding bromide isotopic signals. The exact mass-to-charge detected at 241.008 Th (see the indicated red line) is identified as the cluster of C_7_H_14_O_4_*Br­[79]^−^, and is highlighted with the two probable molecular structures, HP­(a) and HP­(b) within the red dashed box (see Figure 4). The corresponding peak of the bromide isotopic cluster of C_7_H_14_O_4_*Br­[81]^−^ is observed at an exact mass of 243.006 Th (see the blue line). The structure HP­(a) represents hep-trioxy acid that is assumed to form through the hep-APR + OH reaction, analogous to the ben-trioxy acid system as discussed earlier. The structure HP­(b) corresponds to the closed shell hydroxy functionalized product resulting from the Russell mechanism of alkyl peroxy radicals (C_7_H_13_O_5_) formed through the H-shift and O_2_ addition reactions of hep-APR (see the red dashed box in Figure 5).

**Figure 4:**
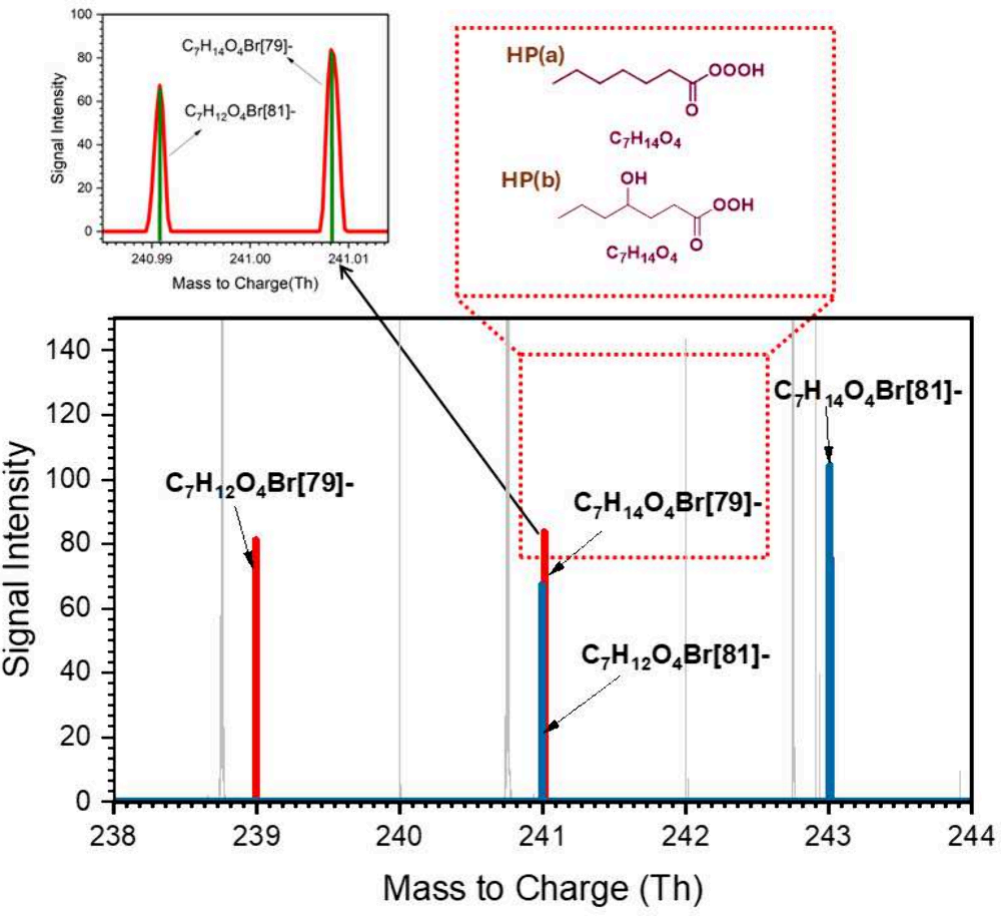
Bromide chemical ionization mass spectrum of O_4_-system (or products) in the OH-initiated heptanaldehyde oxidation. The two potential molecular structures of the molecular formula C_7_H_14_O_4_ are presented within the dashed red box (see Figure 10 in the Supporting Information for formation mechanisms). The zoomed-in spectrum containing the signals of C_7_H_14_O_4_Br­[79]^−^ and C_7_H_12_O_4_Br­[81]^−^ are presented for more clarity. Note: The red and blue peaks are the mass spectra of the corresponding cluster peak with two isotopes of bromide ions, Br[79]- and Br[81]-, respectively.

**Figure 5:**
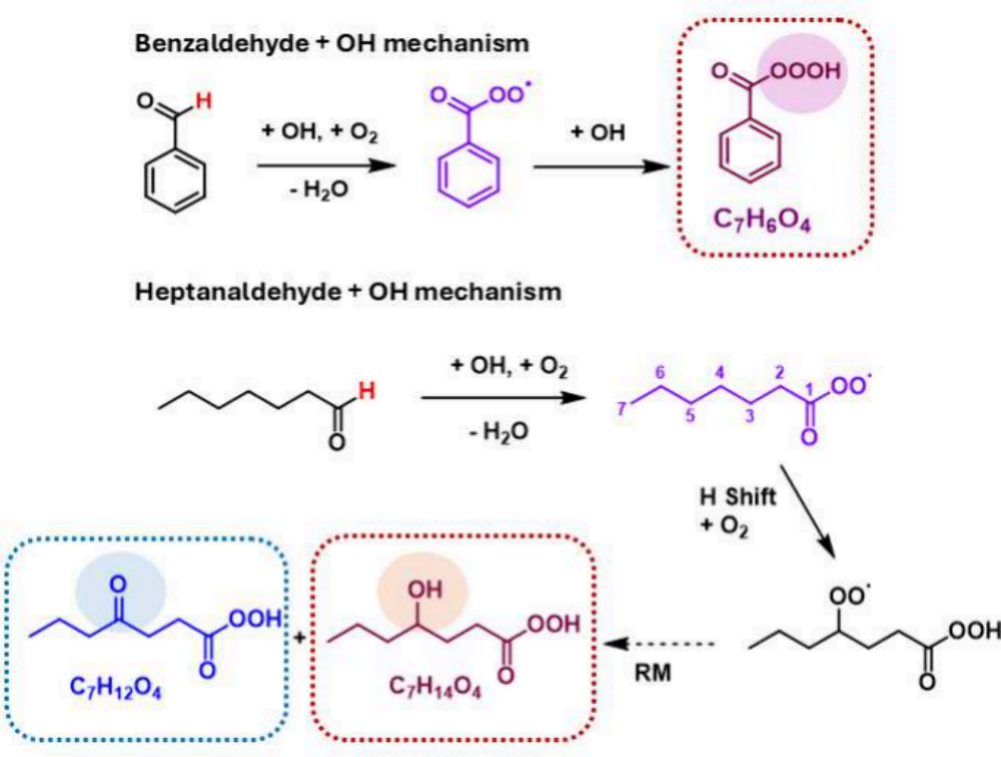
Reaction channels showing ben-trioxy acid formation in the benzaldehyde + OH mechanism, as well as Russell mechanism^17^ (RM) products formed in the heptanaldehyde + OH mechanism, described in this study.

Furthermore, the mass-to-charge ratio at 238.992 Th in mass spectra shown in Figure 4 matches the cluster of C_7_H_12_O_4_*Br­[79]^−^. The chemical composition of C_7_H_12_O_4_ aligns with the other carbonyl product formed in the Russell mechanism (see the blue dashed box in Figure 5). The detailed reaction mechanism of OH-initiated heptanaldehyde oxidation is provided in Figure 10 in the Supporting Information. Previous theoretical results by Seal et al.^11^ and structure–activity relationship (SAR) results from Vereecken and Nozière’s work^10^ indicate that unimolecular hydrogen shift reactions of hep-APR structure are fast with the rate coefficients in the order of 10^–2^ to 10^–1^ s^–1^ for 1,6 H-shift and they constitute the primary channel. Indeed, we observe a two-unit mass shift in our H/D experiments for the signal corresponding to C_7_H_14_O_4_, meaning that the observed product has two labile hydrogen atoms, matching the proposed structure of HP­(b) resulting from the Russell mechanism. Additionally, in order to confirm the Russell mechanism, the corresponding carbonyl compound C_7_H_12_O_4_ must have one labile hydrogen atom, which is indeed confirmed by a one-unit mass shift in H/D experiments. The mass spectra are presented in Figure 12 in the Supporting Information. We did not observe the formation of trioxy acid when using heptanaldehyde, likely due to fast unimolecular reactions of hep-APR. The same can be expected for other APRs with fast unimolecular reactions, meaning that trioxy acid formation is unlikely to occur when unimolecular reactions dominate.

This study highlights the gas-phase reactions of acyl peroxy radicals derived from aldehyde precursors under tropospheric-relevant temperature and pressure. Previously, theoretical studies emphasized the significance of APRs in oxidation chemistry; however, none have described the subsequent unimolecular versus bimolecular channels based on experimental outlook. This study experimentally described the unimolecular and bimolecular chemistry of APRs derived from benzaldehyde and heptanaldehyde. Furthermore, this study indicates that the specific R-group of the aldehyde strongly influences the behavior of APRs. In the case of OH-initiated benzaldehyde oxidation, formation of trioxy acid through the bimolecular reaction with OH radicals is dominant over the unimolecular channel, whereas in the case of OH-initiated heptanaldehyde oxidation, the unimolecular channel outcompetes the bimolecular channel, subsequently producing two stable products through RO_2_ + RO_2_ self-reactions. These reaction channels are illustrated in Figure 5.

The direct detection of trioxy acid derived from the reaction of ben-APR + OH is an instrumental outcome of this study. Nonetheless, the ben-APR + OH pathway is a minor bimolecular channel of ben-APR. Also, the formation of a trioxy acid is not universal with every type of aldehyde precursor, as many APR structures undergo rapid unimolecular reactions. Therefore, understanding the mechanism of trioxy acid formation in atmospheric oxidation chemistry is a challenging task. Lastly, it is important to emphasize the role of the systems containing the -OOOH group, as they could be important for ambient secondary organic aerosol generation, yet currently their involvement in ambient processes is highly uncertain.

## Methodology

We employed a multischeme chemical ionization inlet (MION),^19^ and an Orbitrap mass spectrometer in combination with a quartz flow tube to investigate the OH-initiated oxidation of benzaldehyde and heptanaldehyde. Bromide ions were used as reagent ions, and the residence time in the flow reactor was 1.2 s. The generation of APR in the experimental setup was initiated by introducing the individual aldehyde precursor and hydrogen peroxide (H_2_O_2_) to the flow reactor system by using zero air as a bath gas. The OH radicals were produced by the photolysis of H_2_O_2_ at 254 nm by using a germicidal Hg lamp. All the experiments were conducted at 298 K and 1 atm pressure. The data were analyzed using Orbitool software version 2.3.0.^20^ The details of the experiments performed are provided in the †ESI report (see S1). In addition to the experiments, quantum chemical calculations were employed to understand the bimolecular reaction mechanism of APR + OH, as well as the decomposition of the proposed trioxy acid into the corresponding RO and HO_2_ channels. The theoretical methodology employed in this study is analogous to the previous study by Ahongshangbam et al.^18^

## Supplementary Material



## Data Availability

Experimental files attributed to this study are available from the corresponding author upon request, and the theoretical files are available in a Zenodo repository.

## Notes

Detailed experimental procedure undertaken using flow reactor-Orbitrap-CIMS, possible reaction channels for OH-initiated benzaldehyde and heptanaldehyde oxidation, mass spectrum for PAN experiment, normalized time series plots for all associated systems, H/D experiments results, nitrate-CIMS experimental results, and computational details (PDF)
