# Supplementary material for: Gas-Phase Formation of Trioxy Acid via OH-Initiated Aldehyde Oxidation under Atmospheric Conditions
Source: J Phys Chem Lett. 2026 Jan 16;17(4):1114–8. doi: 10.1021/acs.jpclett.5c03582 (PMC12862793; doi:10.1021/acs.jpclett.5c03582)
Supplement: Supplementary file 1 [file jz5c03582_si_001.pdf]

Supporting Information for: Gas-phase formation of trioxy acid via OH-initiated aldehyde oxidation under atmospheric conditions

Emelda Ahongshangbam,[†,‡] Avinash Kumar,[¶] Shawon Barua,[¶] Melissa Meder,[†,‡] Matti Rissanen,[†,¶] and Nanna Myllys*[,†,‡]

†*Department of Chemistry, University of Helsinki, Helsinki 00014, Finland*

‡*Institute for Atmospheric and Earth System Research, University of Helsinki, Helsinki 00014, Finland*

¶*Aerosol Physics Laboratory, Tampere University, Tampere, 33720, Finland*

E-mail: nanna.myllys@helsinki.fi

Instrumentation and experimental setup

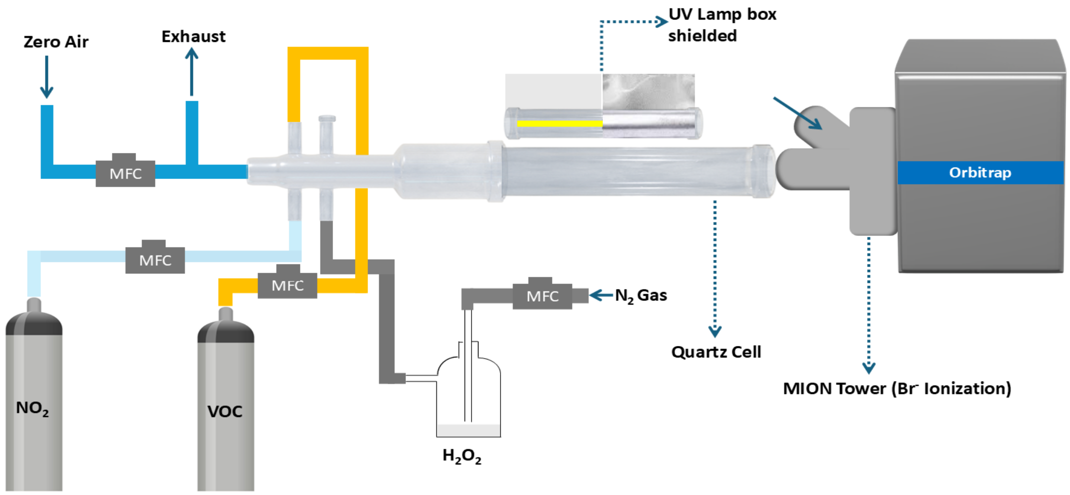

Figure 1: Schematic diagram of the instrument set-up employed in this study.

The experiments were performed in a quartz flow tube reactor (50 cm length and 1 inch OD) coupled to a multischeme chemical ionisation inlet (MION, Karsa Inc.) and an Orbitrap mass spectrometer (Thermo Scientific Exploris 240). Hydroxyl radicals (OH) were produced by photolysing H_2O_2 using a 254 nm germicidal lamp (Osram HNS 15W G13). The products were identified using bromide ionisation, generated in the inlet by exposing dibromomethane (CH_2Br_2) to soft X-rays (Hamamatsu L12535). Clean, dried zero air was used as the bath gas, produced by feeding in-house synthetic air to a zero-air generator (AADCO Instruments, 737 Series). A mist separator (SMC AMG 150C-F01C) and an air membrane dryer (SMC IDG100LA-F04B-P) were connected downstream of the generated zero-air flow for further dehumidification. A total inlet flow of around 14 standard litres per minute (slpm), and the reaction time of 1.2 seconds was maintained throughout the experiments. Calibrated mass flow controllers (Alicat Scientific) were used to control the gas flows inside the flow tube, and the measured values were used to calculate the final concentrations of the reactants in the gas mixture. All experiments were carried out at 298.15 K and 1 atm pressure. The gases were flowed through 6 mm PTFE tubes connected via a series of Swagelok fittings

to the flow reactor. Measurements were also conducted in the presence of nitrogen dioxide (NO_2) to support the formation of acyl peroxy radicals via the corresponding peroxy acyl nitrates (PAN). Hydrogen–deuterium exchange (H/D) experiments were performed in the presence of deuterium oxide (D_2O) to determine the number of replaceable hydrogen atoms (-OH, -OOH, and -OOOH) in the identified products. The mass spectral data obtained in the experiments were analyzed and processed through several steps, including averaging, mass axis calibration, and peak integration in Orbitool software 2.3.0.[1] The concentrations of benzaldehyde, heptanaldehyde, and NO_2 are given in Table 1. The normalised signal intensity of the product X was obtained using the expression given below.

$$\text{Normalized Signal Intensity of } X = \frac{\text{Unnormalized Signal Intensity of } X}{[Br^- + Br[81]^-]\text{reagent ion signal}} \tag{1}$$

Table 1: Concentrations of the experiments used in the experiments. Note: a = The OH radical concentration was inferred from sulfuric acid formation measured by nitrate chemical ionization mass spectrometry. Sulfuric acid was produced via the reaction of SO_2 with OH radicals generated by H_2O_2 photolysis.

Reagent	Concentration (molecule cm^{-3})
Benzaldehyde	(5.3-52.7) * 10^{12}
Heptanaldehyde	(3.1-27.4) * 10^{12}
NO_2	(6.2-35.1) * 10^{11}
OH^a	(5.1-7.2) * 10^{6}

HO_2 radical: The concentration of HO_2 radicals in the reaction system were not estimated. Although HO_2 can be detected by bromide-CIMS[2] under our operating conditions, the instrument was not calibrated for HO_2. Therefore, we report only normalized signal intensities for HO_2 and do not attempt to estimate quantitatively. The normalized intensity of HO_2 radicals in the our experimental condition is 1.077 * 10^{-2}.

The transmission efficiency of the Orbitrap mass spectrometer is mass dependent and may introduce uncertainty in the relative signal intensities of the trioxy acids and other oxygenated products. However, the high mass resolving power (240,000 m/Δm) and mass accuracy enable confident molecular formula assignments. Uncertainties in flow rates de-

rived from mass flow controllers propagate into uncertainties in reactant concentrations and calculated residence times (maximum uncertainty upto 10 %). Wall losses of reactant gases and trioxy-acid products are unavoidable in flow reactors of this size and may reduce absolute signal intensities without affecting product identity. In addition, bromide chemical ionization is sensitive to relative humidity. Introducing D_2O into the reaction mixture may alter the overall relative humidity and influence ionization efficiency. Taken together, these uncertainties primarily affect absolute signal intensities, while the mechanistic conclusions based on product identification and isotopic labeling remain robust.

Chemicals

NO_2 gas cylinder (concentration of 100 ppm in N_2) was obtained from Advanced Speciality gases. UHP Nitrogen gas (5.0 grade) was obtained from Woikoski and Linde Oy. Deuterium oxide (99.9 atom % D) and H_2O_2 (Sigma Aldrich, 50 % wt in H_2O) were obtained from Sigma Aldrich and were bubbled with nitrogen to the flow reactor. Benzaldehyde (ACS reagent, *purity* $\geq$ 99.5%) and heptanaldehyde (ACS reagent, *purity* $\geq$ 99.5%) were commercially obtained from Sigma Aldrich and were supplied from pressurised cylinders containing homemade mixtures of 5.0 N_2 and the reactant aldehyde at ppm levels. All the chemicals were used without further purification.

Probable reaction channels of OH-initiated benzaldehyde oxidation

In Figure 2, we present a detailed probable reaction mechanism of OH-initiated benzaldehyde oxidation. The pink arrows indicate an H-abstraction reaction resulting in the formation of ben-APR (in violet), as well as bimolecular reactions of ben-APR, including the formation of ben-trioxy acid, shown in structure BP(a). Dark violet arrows represent unimolecular

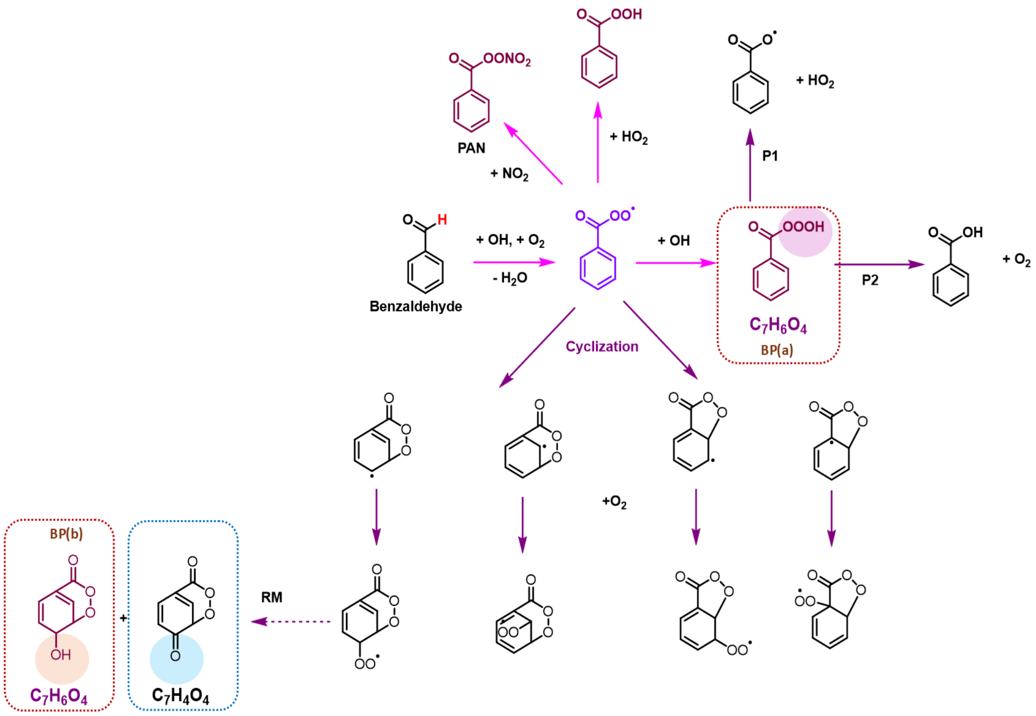

Figure 2: Possible reactions of benzaldehyde + OH in ambient air. The two potential molecular structures of the molecular formula $C_7H_6O_4$ are presented within the dashed red box as BP(a) and BP(b). Note: Structure BP(b) and its corresponding -C=O product are highlighted in red and blue dashed boxes, respectively; however, there could be three additional isomer pairs, resulting from the Russell mechanism of other peroxy radicals presented here.

reactions (endoperoxide ring formations) of ben-APR, which lead to other potential structure BP(b). It is noteworthy that the structure BP(b) has four isomers resulting from different endoperoxide ring-formation mechanisms. The most structurally stable isomer structure is highlighted as a structure BP(b) in the red dashed box. This structure BP(b) is not feasible based on the theoretical studies conducted by Karppinen et al.[3]. The endoperoxide ring formation rates are on the order of 10^{-6} s^{-1} and are shown in Table 2 in the following section. This is because either of the intermediate structures leading to the structure (b) and its corresponding isomers is extremely distorted and strained. Therefore, we selectively present the mass spectra in relevance to structure BP(a) (ben-trioxy acid) and other stable bimolecular products (PAN and ben-peracid).

Although the reactions of ben-trioxy acid with other atmospheric oxidants such as OH,

NO_3 and Cl atoms cannot be ruled out, but their rates are likely to be relatively slow. This can be assumed as hydrogens in ben-trioxy acid are either in an aromatic ring or formed an intramolecular H-bond and there is no non-aromatic C−C double bonds. Thus, both H-abstraction or oxidant addition reactions to the double bond can be assumed to be slow compared to the unimolecular decomposition of ben-trioxy acid.

Confirmation of ben-APR from PAN experiments

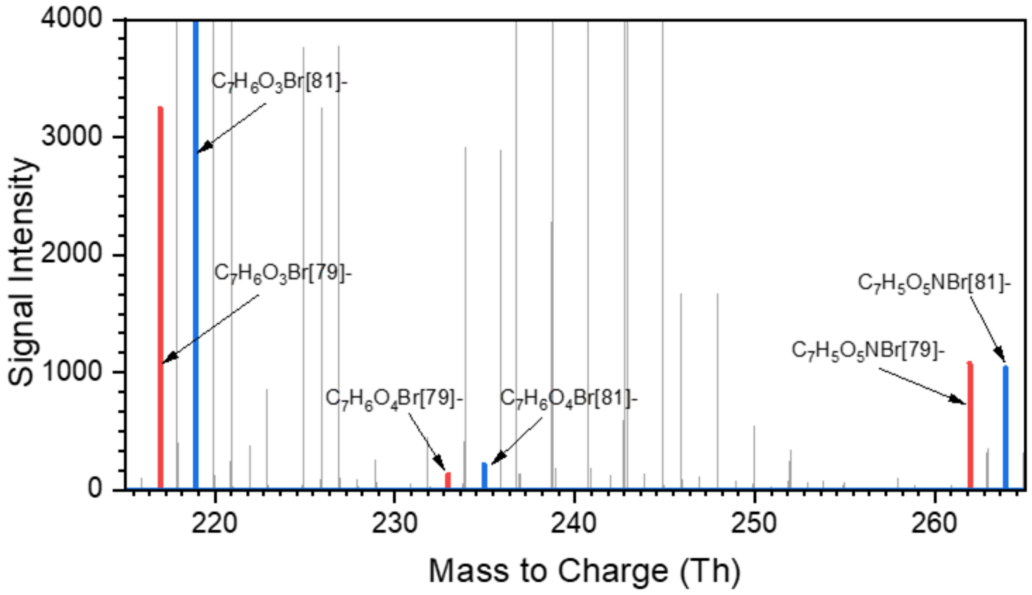

Figure 3: Bromide chemical ionisation mass spectrum showing peroxy acyl nitrate (PAN, $C_7H_5O_3NO_2$) in ben-APR + NO_2 reactions and other bimolecular reaction products. The red and blue peaks are the mass spectra of the corresponding cluster peak with two isotopes of bromide ions, Br[79]- and Br[81]-, respectively and the grey peaks represent additional peaks which are not in the focus of this study. Note: In this mass spectrum, the concentration of benzaldehyde is $7.9 * 10^{12}$ molecules cm^{-3} and that of NO_2 is $3.5 * 10^{12}$ molecules cm^{-3}.

Based on the main findings of this study, specifically in the formation of ben-trioxy acid, the initial step of ben-APR indicates that bimolecular reactions are the dominant pathway over the unimolecular channel under low NOx conditions. To support this finding, we also examine the OH-initiated benzaldehyde oxidation under the previously stated conditions in

the presence of 143 ppb of NO_2 in the flow reactor. The idea is that when NO_2 is added, the APR is converted to the corresponding PAN species that can be detected, thereby supporting the assignment of $C_7H_5O_3$ as a ben-APR. In the mass spectrum in Figure 3, we observe the signal intensity corresponding to ben-peroxyl acyl nitrate cluster with $Br[79]^-$ with a molecular formula $C_7H_5O_5NBr^-$ at mass-to-charge 261.935 Th (in red color), along with its bromide isotopic cluster peak at 263.933 Th (in blue color). Another observation highlights the presence of ben-trioxy acid ($C_7H_6O_4$) with a lesser intensity due to the high NOx condition, where ben-PAN formation channel outcompetes this trioxy acid formation channel. Moreover, the mass-to-charge ratio at 216.950 Th, along with the corresponding bromide isotopic cluster peak at 218.948 Th, with a molecular formula $C_7H_6O_3Br[79]^-$ and $C_7H_6O_3Br[81]^-$, respectively, matches the closed-shell product containing three oxygen atoms. This peak likely arises from the ben-APR + HO_2 mechanism, resulting in the formation of ben-peracid ($C_7H_6O_3$). Large difference in the signal intensity between the $C_7H_6O_4$ and $C_7H_6O_3$ products (see Figure 3) is likely due to the higher concentration of HO_2 than OH. To further address this chemistry, we specifically include the ben-peracid, $C_7H_6O_3$ in the time series plot of ben-trioxy formation and in the mass spectrum of the H/D exchange experiment (see Figure 5 and 6). The detailed chemistry, based on the large intensity of the $C_7H_6O_3$ system relative to the other bimolecular channels, is explained in the following section.

Time series of OH-initiated benzaldehyde oxidation

The normalised time series plots of the formation of ben-trioxy acid and ben-peracid are presented in Figures 4 and 5, respectively. The bold lines correspond to the main cluster peak, and those of the dashed lines are for their respective bromide isotopic peak. The trends are consistent with the concentration of benzaldehyde in the flow tube reactor. We initiated with a concentration of 5.3 x 10^{12} cm^{-3} of benzaldehyde, followed by $7.9*10^{12}$ cm^{-3}, $9.2*10^{12}$

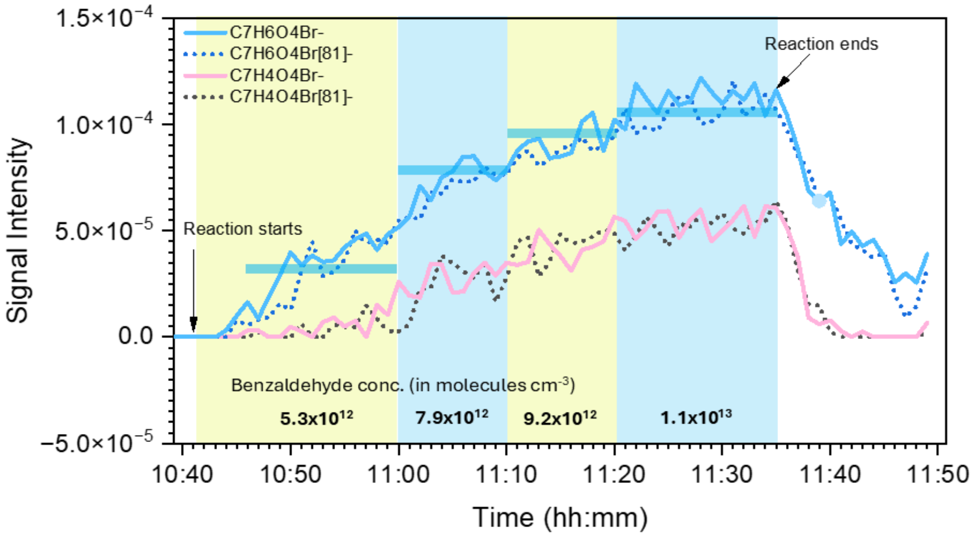

Figure 4: Normalised time series plot for the O_4-products in the OH-initiated benzaldehyde oxidation. Note: The ben-trioxy acid ($C_7H_6O_4Br[79]^-$) is shown in a blue line along with the horizontal blue bars showing the average signal intensity in correspondence to the benzaldehyde concentration. The colored regions indicatGas the benzaldehyde concentrations (in molecules cm^{-3}).

cm^{-3} and $1.1 * 10^{13}$ cm^{-3}. The mass spectrum presented in the main paper for the trioxy acid formation is from the average mass spectra of 15 minutes, when the concentration of benzaldehyde is $1.1 * 10^{13}$ cm^{-3} and that of H_2O_2 is between $1.5 - 1.8 * 10^{15}$ cm^{-3}. Moreover, in Figure 5, the trend for the $C_7H_6O_3$-system aligns well with the time series plot of ben-trioxy acid (shown in Figure 4), however, the signal intensity was significantly higher in comparison to that of ben-trioxy acid. Thus, it suggests that, in addition to the ben-APR + HO_2 product ($C_7H_6O_3$), there could be a significant presence of another stable product with the same molecular formula, $C_7H_6O_3$, with salicylic acid as the proposed candidate. To investigate this possibility, we inspect the structure of this composition by analyzing the number of -OH groups through the hydrogen/deuterium exchange reaction. The details of this analysis are given in the following section.

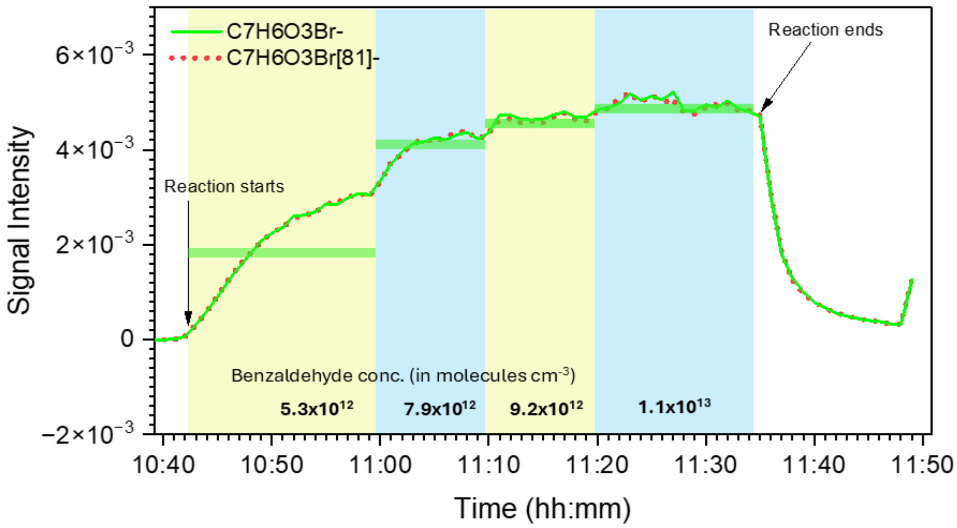

Figure 5: Normalised time series plot for the $C_7H_6O_3$-product in the OH-initiated benzaldehyde oxidation. Note: The compound ($C_7H_6O_3Br[79]^-$) is shown in a light green line along with the horizontal green bars showing the average signal intensity in correspondence to the benzaldehyde concentration. The colored regions indicate the benzaldehyde concentrations (in molecules cm^{-3}).

Mass spectra from deuterium-exchanged experiments

This experiment is based on simple chemistry, where deuterium atoms in the heavy water replace weakly bound hydrogen atoms in a molecule. The number of H/D shift indicate the number of -OH, -OOH, or -OOOH functional groups in a given molecule.

Mass spectra of $C_7H_6O_3$ from the deuterated experiments. Figure 6 shows that there exist two mass signals with varying deuterium atom numbers, as well as with different signal intensities. The first red color signal at the mass-to-charge ratio at 217.956 Th corresponds to the chemical formula of a bromide cluster ($C_7H_5D_1O_3Br[79]^-$), while the one in blue color shows the bromide isotopic cluster ($C_7H_5D_1O_3Br[81]^-$) at the mass-to-charge ratio 219.954 Th. The presence of one deuterium atom, particularly in this chemical for-

mula, signals the presence of one weakly bound hydrogen atom, supporting the detection of ben-peracid as indicated by a molecular structure in the mass spectrum (see Figure 6). Additionally, signals at 218.963 Th and 220.961 Th align with the cluster of ($C_7H_4D_2O_3Br[79]^-$) and its isotopic cluster ($C_7H_4D_2O_3Br[81]^-$). Interestingly, these signals are quite intense, and the presence of two deuterium atoms indicates the molecule possesses two labile hydrogen atoms, and based on these observations, the formation of salicylic acid (as depicted in the mass spectrum in Figure 6) can be suggested. The formation of salicylic acid from the OH-initiated oxidation of benzaldehyde at atmospheric temperature and pressure is not the core focus of this study, but it highlights that unexplored mechanisms of benzaldehyde oxidation still exist.

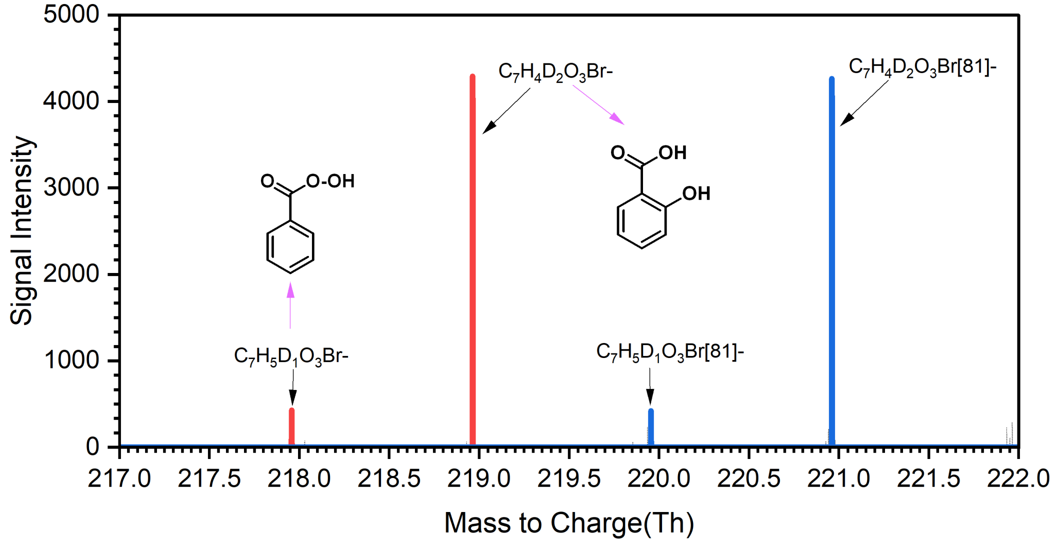

Figure 6: Bromide chemical ionisation mass spectrum of OH-initiated benzaldehyde oxidation reaction in the presence of D_2O, illustrating the formation of ben-peracid (via $C_7H_5D_1O_3Br^-$) and suggested structure of salicylic acid (via $C_7H_4D_2O_3Br^-$). Note: The red and blue peaks are the mass spectra of the corresponding cluster peak with two isotopes of bromide ions, Br[79]- and Br[81]-, respectively.

Mass spectra of $C_7H_6O_4$ and $C_7H_4O_4$ from the deuterated experiments. As seen in the mass spectrum plot shown in Figure 7, there is a mass signal at 233.951 Th (in red color) and another one at 235.949 Th (in blue color), aligning with the masses of

clusters of $C_7H_5D_1O_4Br[79]^-$ and that of $C_7H_5D_1O_4Br[81]^-$, respectively. This shows that the chemical formula of $C_7H_6O_4$ possesses only one replaceable hydrogen, suggesting that the hydrogen atom could be from the -OH or -OOOH group from two potential structures BP(a) and BP(b) (refer to Figure 2). However, as shown in Figure 7, $C_7H_4O_4$ exhibits one H/D shift, indicating the presence of one -OH group. This observation is in contrast to the $C_7H_4O_4$ formed through the Russell mechanism, which has no-OH group (refer to Figure 2 for structure). Thus, it implies that these mass signals are not relevant to the Russell mechanism products, and $C_7H_6O_4$ instead corresponds to ben-trioxy acid product.

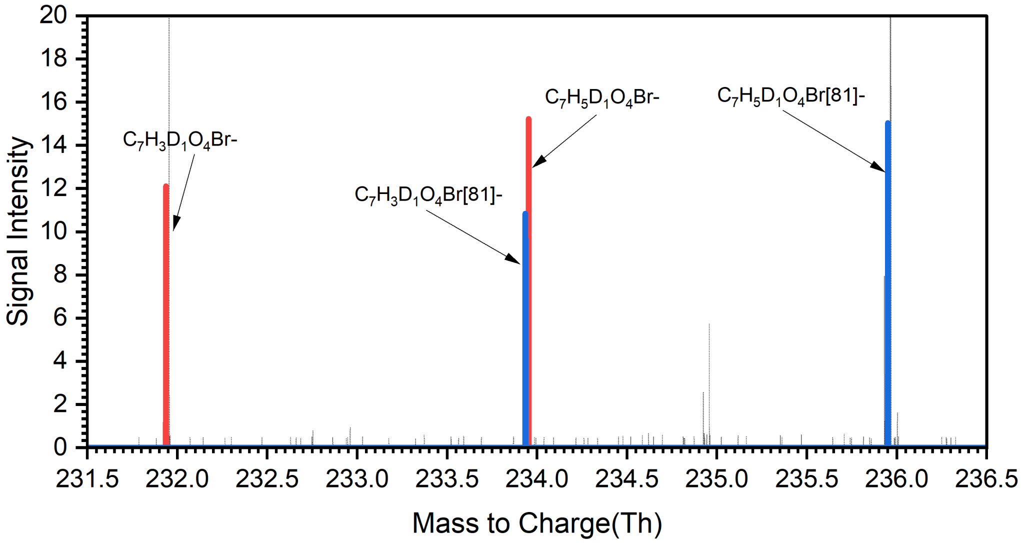

Figure 7: Mass spectrum of OH-initiated benzaldehyde oxidation reaction in the presence of D_2O, illustrating the formation of ben-trioxy acid (via $C_7H_5D_1O_4Br^-$). Note: The red and blue peaks are the mass spectra of the corresponding cluster peak with two isotopes of bromide ions, Br[79]- and Br[81]-, respectively.

Ben-trioxy acid detection by nitrate-CIMS

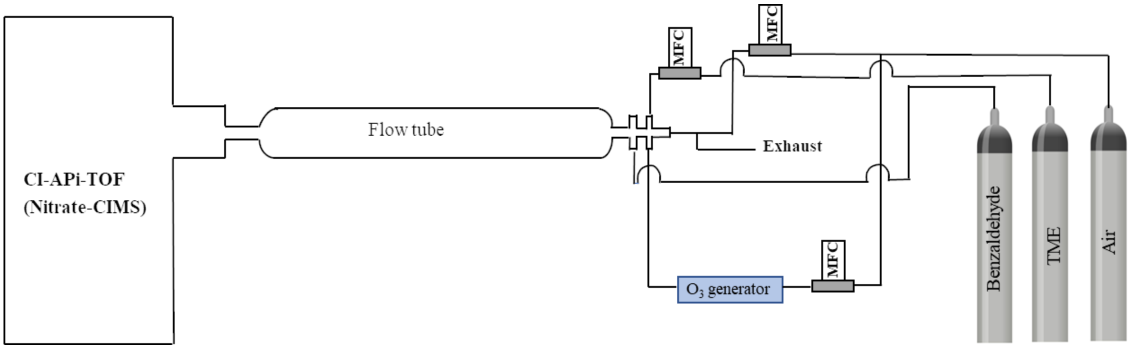

Figure 8: A nitrate ($NO_3{}^-$) based chemical ionisation mass spectrometer coupled to an atmospheric pressure flow reactor. The oxidant OH radical is produced in situ by TME + O_3 reaction. TME = tetramethylethylene. All flows are controlled by mass flow controllers (MFCs).

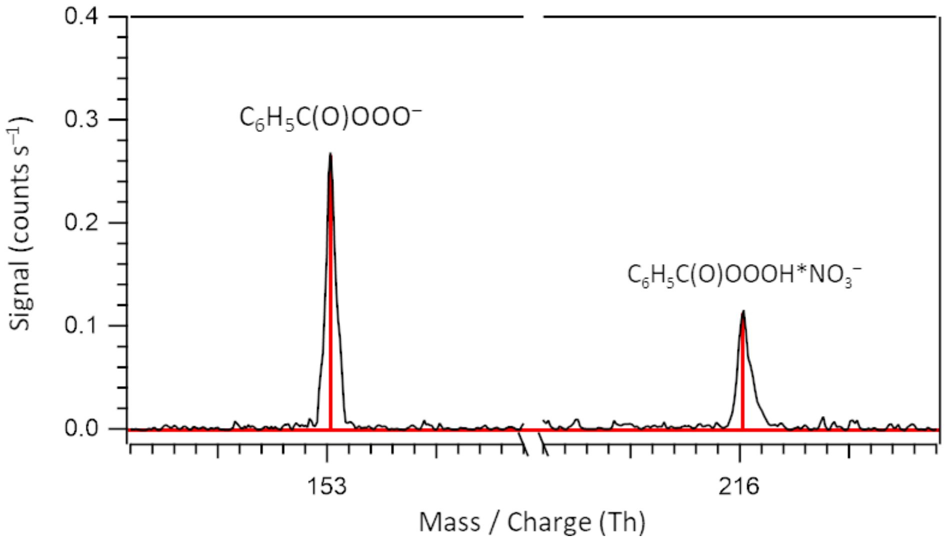

Figure 9: Nitrate-CIMS spectrum showing the detection of ben-trioxy acid as $NO_3{}^-$ adduct ($C_6H_5C(O)OOH \cdot NO_3{}^-$) and its deprotonated signal ($C_6H_5C(O)OO^-$).

In addition to MION-Orbitrap mass spectrometry detection as discussed earlier, the ben-trioxy acid is also detected using a nitrate-based chemical ionisation atmospheric pressure interface time-of-flight mass spectrometer ($NO_3{}^-$-CI-APi-TOF), known as nitrate-CIMS. The OH-initiated benzaldehyde oxidation reaction was conducted using a borosilicate flow reactor setup (see Figure 8). A sheath flow of 20 slpm to the Eisele CI inlet, and flow reactor bath gas maintaining a total sample flow of 8.1 slpm were supplied by the zero-air

generator (AADCO-737-15). X-ray was used to produce NO_3^- from gas-phase nitric acid (HNO_3) carried by N_2 that mixed with the sheath flow. Benzaldehyde was supplied to the reactor from a gas cylinder while the oxidant hydroxyl radical (OH) was produced in situ by the ozonolysis of tetramethylethylene (TME). Ozone was produced by photolysis of clean air using a mercury lamp (UVP, Analytik Jena), and its concentration was measured using an ozone analyser (2B Technologies model 205). The mass spectrometer inlet flow (8.1 lpm) and the volume of the reactor (1 m length and 4.7 cm inner diameter) define the residence time of the reaction mixture inside the reactor to be 14 s. In this experiment, we observe the formation of ben-APR-derived trioxy acid ($C_7H_6O_4$) in terms of its adduct with NO_3^- as well as its deprotonated form ($C_7H_5O_4^-$) by H^+ transfer to the NO_3^- as shown in Figure 9. The lower signal intensity of the trioxy acid compared to its deprotonated anion can be due to the poor clustering energy with NO_3^- for species with less than two hydrogen bonding functional groups.[4]

Probable reaction channels of OH-initiated heptanaldehyde oxidation

We also explore the probable reaction mechanisms of the OH-initiated heptanaldehyde oxidation reaction. Similarly to the reaction mechanism of benzaldehyde oxidation (shown in Figure 10), the mechanisms presented here also include the competitive reactions between bimolecular reactions with other oxidants (OH, HO_2) as well as unimolecular reactions associated with hep-APR. It is noteworthy to mention that the chemical formula of the expected hep-trioxy acid is $C_7H_{14}O_4$, and is shown HP(a) in a red dashed box, and is also equivalent to the chemical formula of structure HP(b) (shown in red dashed box) formed via unimolecular H-shift and O_2 addition of hep-APR, succeeded by self-reaction through the Russell mechanism. It is important to note that another structure (say -C=O product) is also produced through the same Russell mechanism, with a chemical formula $C_7H_{12}O_4$ in an equal

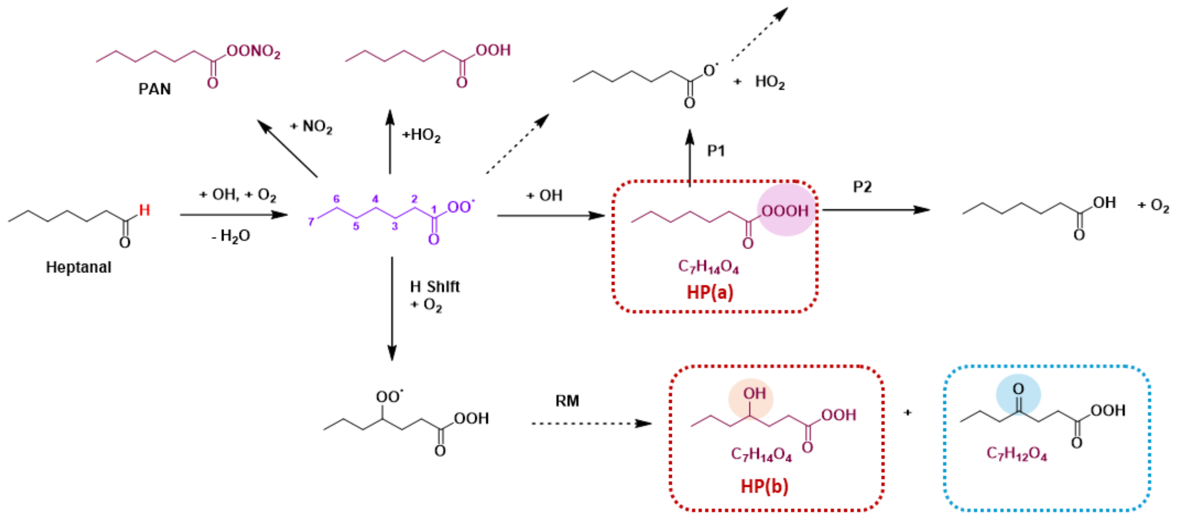

Figure 10: Probable reactions of heptanaldehyde + OH in ambient air. The two potential molecular structures of the molecular formula $C_7H_{14}O_4$ are presented within the dashed red box.

concentration with HP(b). This -C=O product is indicated in a blue dashed box in Figure 10 for clarity. Thus, it is crucial to inspect the mass spectra for both structures by adding D_2O for accurate confirmation. A comprehensive theoretical analysis was conducted by Seal et al.[5] about the hydrogen shift reactions of various acyl peroxyl radical systems originating from a range of aliphatic aldehyde precursors. All the energy barriers and rate coefficients for all the hep-APR H-shifts are listed under the computational details section of this ESI (refer to Table 2).

Time series of OH-initiated heptanaldehyde oxidation

The normalised time series plot is presented in Figure 11 as a reference to the mass spectrum shown in the main paper (see Figure 4) concerning the hep-derived product. The bold lines correspond to the main cluster peak with bromide, and the dashed lines illustrate the cluster associated with the isotopic peak of the bromide reagent ion. It can be seen that both the

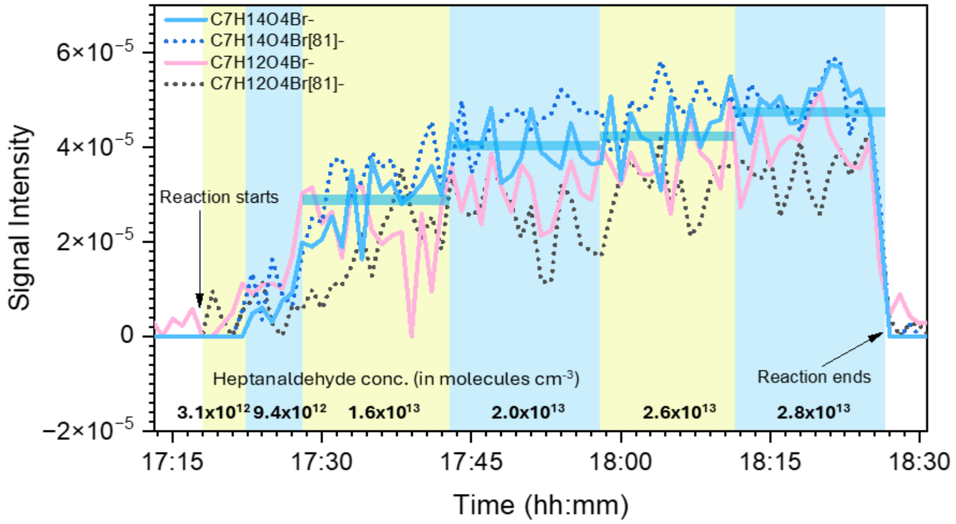

Figure 11: Normalised time series plot for the O_4-product in the OH-initiated heptanaldehyde oxidation. Note: The closed-shell compound ($C_7H_{14}O_4Br[79]^-$) is shown in a blue line along with the horizontal blue bars showing the average signal intensity in correspondence to the heptanaldehyde concentration. The colored regions indicate the heptanaldehyde concentrations (in molecules cm^{-3}).

clusters ($C_7H_{14}O_4$ and $C_7H_{12}O_4$), i.e HP(b) and -C=O product as described earlier, appear to form with a comparable intensity and change accordingly to the varying concentration of heptanaldehyde in the system. This strongly suggests the $C_7H_{14}O_4$ and $C_7H_{12}O_4$ products are coming from the Russell mechanism. It is noteworthy that the heptanaldehyde concentration was $3.1 * 10^{12}$ cm^{-3} during injection, followed by $9.4 * 10^{12}$ cm^{-3}, at which point the signal intensity began to become prominent. The concentration was increased gradually until it reached $2.8 * 10^{13}$ cm^{-3}. The average mass spectrum shown in the main paper was from the reaction when the heptanaldehyde concentration was $2.8 * 10^{13}$ cm^{-3} and that of H_2O_2 flow was between $1.5 - 1.8 * 10^{15}$ cm^{-3}.

Mass spectrum from hydrogen/deuterium exchange experiments

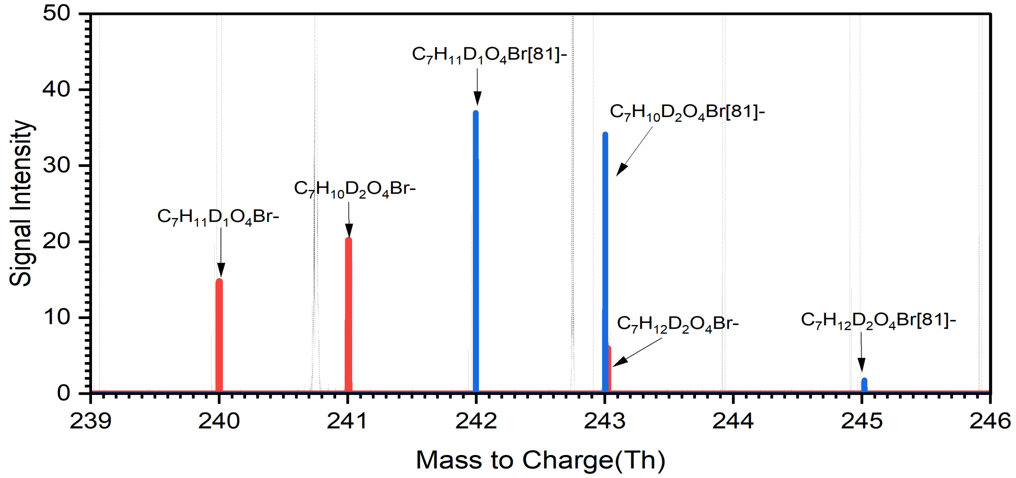

Figure 12: Bromide chemical ionisation mass spectrum showing $C_7H_{14}O_4$-product with 2 labile hydrogens in OH-initiated heptanaldehyde oxidation reaction in the presence of D_2O. Note: There is a $C_7H_{12}O_4$ compound (-C=O product) with 1 and 2 labile hydrogens, and we have only identified the structure with 1 labile hydrogen.

Building upon the benzaldehyde oxidation studies in the presence of D_2O, we further explore the verification of HP(b) and -C=O product in the presence of D_2O. The goal is to

identify the mass spectral signals corresponding to the molecular formula $C_7H_{12}D_2O_4$, and $C_7H_{11}D_1O_4$, which are the representatives of parent compounds containing two –OH groups ($C_7H_{14}O_4$) and a single –OH group ($C_7H_{12}O_4$), respectively. Interestingly, a mass-to-charge ratio at 243.020 Th, in the mass spectrum plot in Figure 12, has two mass units greater than the mass-to-charge ratio of the cluster of $C_7H_{14}O_4 \cdot Br[79]^-$, indicating the presence of one -OH and one -OOH groups. Also, we do not observe any bromide cluster corresponding to $C_7H_{13}D_1O_4$, which is the structure having one -OOOH group and is aligned to HP(a) structure. Thus, we confirm that the $C_7H_{14}O_4$ is an HP(b) structure. Additionally, the observation of $C_7H_{11}D_1O_4Br^-$ shows that the structure has one -OH group, further substantiating the presence of a -C=O product that forms alongside the HP(b) in the unimolecular reaction of hep-APR.

Computational details

Table 2: Energy barrier (in kcal mol^{-1}) with their corresponding rate coefficients (in s^{-1}) for all the reaction mechanisms succeeding the acyl peroxyl radicals in the OH-initiated aldehyde oxidation reactions. Note: This work[a] corresponds to the association rate (in cm^3 molecule^{-1} s^{-1}) of the reaction ben-APR + OH calculated using long-range transition state theory,[6] and the work[b] corresponds to the dissociation rate coefficient (in s^{-1}) calculated using the master equation solver for multi energy-well reactions (MESMER) software.[7]

Reactions	Energy barrier	Rate Coefficients	Reference
Ben-APR+OH → Ben-trioxy acid	Barrierless	$8.0 * 10^{-10}$	This work[a]
Ben-APR (1,5 H-shift)	29.8	$2.6 * 10^{-9}$	Karppinen et al.[3]'s work
Ben-APR (4-endoperoxide)	24.8	$1.9 * 10^{-6}$	”
Ben-APR (5-endoperoxide)	23.5	$5.7 * 10^{-6}$	”
Ben-trioxy acid →Ben-alkoxy + HO_2	Barrierless	$2.2 * 10^{-1}$	This work[b]
Ben-trioxy acid →Ben-acid + $^1O_2$	16.2	$3.7 * 10^{1}$	Chen et al.[8]'s work
Hep-APR (1,4 H-shift)	27.0	$8.9 * 10^{-7}$	Seal et al.[5]'s work
Hep-APR (1,5 H-shift)	20.9	$1.0 * 10^{-2}$	”
Hep-APR (1,6 H-shift)	19.0	$2.4 * 10^{-1}$	”
Hep-APR (1,7 H-shift)	17.4	$1.5 * 10^{-1}$	”
Hep-APR (1,8 H-shift)	18.7	$2.0 * 10^{-2}$	”
Hep-APR (1,9 H-shift)	20.9	$4.2 * 10^{-4}$	”

To corroborate the experimental findings of this study, we incorporate quantum chemistry to investigate the potential reaction channels as described in the Figure 2 and Figure 10. Most of the potential channels have already been theoretically studied;[3,5,8] therefore, we report the values of barrier heights (in kcal mol^{-1}) and rate coefficients (in s^{-1}) for each channel based on these references given in Table 2. However, we performed the calculations for the association of ben-APR and OH radicals using long-range transition state theory[?] as well as the decomposition reactions associated with the ben-trioxy acid using the master equation solver for multi-energy-well reactions (MESMER) software.[7] The values of 4.5 and 602.7 were used as the Lennard-Jones parameters σ (Å) and ε/k_B (K), respectively, for MESMER calculations, and the relative electronic energies (R.E) of 36.79 and 26.49 in kcal mol^{-1} were obtained for ben−APR + OH and ben−alkoxy + HO_2 reactions, respectively. All calculations were conducted at the DLPNO–CCSD(T)/aug-cc-pVTZ[9–11]//ωB97X-D[12]/6-31+G*[13,14] level of theory. This method is analogous to the previous study employed by Ahongshangbam et al.[15] regarding the decomposition pathways of isoprene-derived hydrotrioxide in the atmosphere.

Table 2 shows that the association reaction of ben-APR + OH is very rapid, forming ben-trioxy with no energy barrier. The reaction proceeds via the formation of a pre-reactive complex (PRC), and is analogous to the reaction of RO_2+OH mechanism. Several studies also agree with the barrierless formation of trioxides in the reaction of RO_2+OH.[12,16,17] Moreover, based on the energy barriers and rate coefficients, all the unimolecular channels of ben-APR are slow and in excellent agreement with the experimental results of this study. However, Chen et al.[8]'s take on the decomposition of the trioxy acid into its corresponding acid and singlet molecular oxygen via the HAT mechanism does not align with the experimental finding of this study. We agree that the mechanism is more relevant for the smaller R-group, such as acetaldehyde. However, we are skeptical of this finding in the case of benzaldehyde-derived trioxy acid, as the TS structure breaks complementary ring formation in the molecule. Berndt et al.[17] also showed that the gas phase decomposition into the singlet O_2 is unlikely to occur in the atmosphere. Nevertheless, our main experimental finding,

highlighting the formation of trioxy acid derived from benzaldehyde, sheds the importance of the type of R-group constituting APR on proceeding further APR+OH reaction channels.

The results of heptanaldehyde-derived APR H-shift mechanisms have been highlighted based on the theoretical study by Seal et al.[5]. We specifically present all the H-shifts to provide a comprehensive understanding of the significant rates of hydrogen migration, which depend on the position within the carbon chain. The 1,6 and 1,5 H-shifts are the fastest with rate coefficients in order of 10^{-1} s^{-1}. This is also quite in agreement with the similar study by Barua et al.[18] for hexanaldehyde-APR hydrogen shift reactions. This concludes that for the aliphatic chain aldehyde, the most optimal position of the radical centre lies nearly around the fourth carbon from the carbonyl centre of the chain.

Table 3: Selected vibrational frequencies (in cm^{-1}) and their corresponding intensities in the computed infrared spectrum of ben-trioxy acid (refer Figure 3 in the main text for molecular structure).

Frequency	Intensity	Assignment
860.31	103.86	-O-O- stretch
1007.93	58.98	-O-O- stretch (H-bonded)
1296.82	321.62	-C-O stretch
1492.42	101.93	-OH bend
1833.44	260.64	-C=O stretch
3668.46	47.37	-OH stretch

References

(1) Cai, R.; Li, Y.; Clément, Y.; Li, D.; Dubois, C.; Fabre, M.; Besson, L.; Perrier, S.; George, C.; Ehn, M. et al. Orbitool: a software tool for analyzing online Orbitrap mass spectrometry data. *Atmospheric Measurement Techniques* **2021**, *14*, 2377–2387.

(2) He, X.-C.; Shen, J.; Iyer, S.; Juuti, P.; Zhang, J.; Koirala, M.; Kytökari, M. M.; Worsnop, D. R.; Rissanen, M.; Kulmala, M. et al. Characterisation of gaseous iodine species detection using the multi-scheme chemical ionisation inlet 2 with bromide and nitrate chemical ionisation methods. *Atmospheric Measurement Techniques* **2023**, *16*, 4461–4487.

(3) Karppinen, I.; Pasik, D.; Ahongshangbam, E.; Myllys, N. The impact of unimolecular reactions on acyl peroxy radical initiated isoprene oxidation. *Aerosol Research* **2025**, *3*, 175–183.

(4) Hyttinen, N.; Kupiainen-Maatta, O.; Rissanen, M. P.; Muuronen, M.; Ehn, M.; Kurtén, T. Modeling the charging of highly oxidized cyclohexene ozonolysis products using nitrate-based chemical ionization. *The Journal of Physical Chemistry A* **2015**, *119*, 6339–6345.

(5) Seal, P.; Barua, S.; Iyer, S.; Kumar, A.; Rissanen, M. A systematic study on the kinetics of H-shift reactions in pristine acyl peroxy radicals. *Physical Chemistry Chemical Physics* **2023**, *25*, 28205–28212.

(6) Georgievskii, Y.; Klippenstein, S. J. Long-range transition state theory. *The Journal of Chemical Physics* **2005**, *122*.

(7) Glowacki, D. R.; Liang, C.-H.; Morley, C.; Pilling, M. J.; Robertson, S. H. MESMER: An Open-Source Master Equation Solver for Multi-Energy Well Reactions. *The Journal of Physical Chemistry A* **2012**, *116*, 9545–9560.

(8) Chen, H.; Wang, S.; Wang, L. Reaction of the Acetyl Peroxy Radical and OH Radical as a Source of Acetic Acid in the Atmosphere. *ACS Earth and Space Chemistry* **2024**, *8*, 2522–2531.

(9) Kendall, R. A.; Dunning, T. H.; Harrison, R. J. Electron affinities of the first-row atoms revisited. Systematic basis sets and wave functions. *The Journal of chemical physics* **1992**, *96*, 6796–6806.

(10) Myllys, N.; Elm, J.; Halonen, R.; Kurtén, T.; Vehkamäki, H. Coupled cluster evaluation of the stability of atmospheric acid–base clusters with up to 10 molecules. *J. Phys. Chem. A* **2016**, *120*, 621–630.

(11) Guo, Y.; Riplinger, C.; Becker, U.; Liakos, D. G.; Minenkov, Y.; Cavallo, L.; Neese, F. Communication: An improved linear scaling perturbative triples correction for the domain based local pair-natural orbital based singles and doubles coupled cluster method [DLPNO-CCSD(T)]. *The Journal of chemical physics* **2018**, *148*, placeholder.

(12) Chai, J.-D.; Head-Gordon, M. Long-range corrected hybrid density functionals with damped atom–atom dispersion corrections. *Physical Chemistry Chemical Physics* **2008**, *10*, 6615.

(13) Clark, T.; Chandrasekhar, J.; Spitznagel, G. W.; Schleyer, P. V. R. Efficient diffuse function-augmented basis sets for anion calculations. III. The 3-21+G basis set for first-row elements, Li–F. *Journal of Computational Chemistry* **1983**, *4*, 294–301.

(14) Hehre, W. J.; Ditchfield, R.; Pople, J. A. Self—Consistent Molecular Orbital Methods. XII. Further Extensions of Gaussian—Type Basis Sets for Use in Molecular Orbital Studies of Organic Molecules. *The Journal of Chemical Physics* **1972**, *56*, 2257–2261.

(15) Ahongshangbam, E.; Franzon, L.; Almeida, T. G.; Hasan, G.; Frandsen, B. N.; Myllys, N. Decomposition pathways of isoprene-derived hydrotrioxides and their clus-

tering abilities in the atmosphere. *Physical Chemistry Chemical Physics* **2025**, *27*, 5889–5901.

(16) Müller, J.-F.; Liu, Z.; Nguyen, V. S.; Stavrakou, T.; Harvey, J. N.; Peeters, J. The reaction of methyl peroxy and hydroxyl radicals as a major source of atmospheric methanol. *Nature Communications* **2016**, *7*.

(17) Berndt, T.; Chen, J.; Kjærgaard, E. R.; Møller, K. H.; Tilgner, A.; Hoffmann, E. H.; Herrmann, H.; Crounse, J. D.; Wennberg, P. O.; Kjaergaard, H. G. Hydrotrioxide (ROOOH) formation in the atmosphere. *Science* **2022**, *376*, 979–982.

(18) Barua, S.; Iyer, S.; Kumar, A.; Seal, P.; Rissanen, M. An aldehyde as a rapid source of secondary aerosol precursors: theoretical and experimental study of hexanal autoxidation. *Atmospheric Chemistry and Physics* **2023**, *23*, 10517–10532.
